# *Drosophila* female reproductive glands contribute to mating plug composition and the timing of sperm ejection

**DOI:** 10.1098/rspb.2021.2213

**Published:** 2022-02-09

**Authors:** Caitlin E. McDonough-Goldstein, Scott Pitnick, Steve Dorus

**Affiliations:** Center for Reproductive Evolution, Biology Department, Syracuse University, Syracuse, NY 13244, USA

**Keywords:** spermatheca, parovaria, mating plug, sperm ejection, ejaculate-female interactions, female reproductive tract

## Abstract

Reproductive traits that influence female remating and competitive fertilization rapidly evolve in response to sexual selection and sexual conflict. One such trait, observed across diverse animal taxa, is the formation of a structural plug inside the female reproductive tract (FRT), either during or shortly after mating. In *Drosophila melanogaster*, male seminal fluid forms a mating plug inside the female bursa, which has been demonstrated to influence sperm entry into storage and latency of female remating. Processing of the plug, including its eventual ejection from the female's reproductive tract, influences the competitive fertilization success of her mates and is mediated by female × male genotypic interactions. However, female contributions to plug formation and processing have received limited attention. Using developmental mutants that lack glandular FRT tissues, we reveal that these tissues are essential for mating plug ejection. We further use proteomics to demonstrate that female glandular proteins, and especially proteolytic enzymes, contribute to mating plug composition and have a widespread impact on plug formation and composition. Together, these phenotypic and molecular data identify female contributions to intersexual interactions that are a potential mechanism of post-copulatory sexual selection.

## 1. Introduction

Functional interactions between male and female reproductive traits are intrinsic to most major theoretical models of sexual selection addressing intersexual choice (e.g. good genes, runaway selection, sensory exploitation, sexually antagonistic coevolution, genetic incompatibility; [1–6]. These interactions are credited with the widespread pattern of rapid evolutionary co-diversification of interacting sex-specific traits [7–9]. However, our knowledge of the mechanisms of intersexual interaction that are required to fully understand variation in fitness remains limited.

Resolving precise mechanisms of intersexual interaction is likely to be more attainable for postcopulatory sexual selection, occurring whenever females remate within a reproductive cycle [10,11], as the relevant male traits (e.g. genitalic morphology, sperm form, seminal fluid composition) interact directly with the female reproductive tract (FRT) [9,11–14]. Moreover, recent theoretical and empirical contributions suggest that postcopulatory intrasexual competition (i.e. sperm competition) and intersexual choice (i.e. cryptic female choice) are a false dichotomy [15–18]. Hence, virtually all reproductive processes contributing to variation in competitive fertilization success are likely to involve some female × male interactions (e.g. [19–26]). Postcopulatory sexual selection theory has thus been transitioning over the past few decades away from the historical male bias in traits of interest and the presumption of exclusive male agency, and moving towards a theoretical framework that recognizes the critical contribution of both females and males to interactions that influence the expression of complex traits and reproductive outcomes [27–29].

A prime example of a taxonomically widespread trait that has historically been viewed as strictly male-mediated is the formation of structured ejaculates, such as spermatophores and mating plugs [30,31]. This long-held presumption, however, was recently disproved in an investigation of the cabbage white butterfly, *Pieris rapae*, which revealed that newly formed spermatophores contain large quantities of female-derived proteases [32]. Moreover, isotopic labelling and proteomics identified six candidate female-derived proteins in the mating plugs of the house mouse, *Mus domesticus* [33]. These examples suggest a potentially widespread, active role of female contribution to these structures.

Mating plugs have independently evolved numerous times in diverse taxa [31] and have been shown to prevent remating or limit the transfer and/or transport of sperm by future mates of the female (e.g. [34–36]). The formation of a large mating plug in the FRT is a critical component of reproduction in the fruit fly *Drosophila melanogaster* [37] in which its principal selective functions appear to be facilitating efficient sperm storage [38] and mediating postcopulatory sexual selection through the timing of sperm ejection [17,37,39–42]. Specifically, longer retention of the plug provides more time for sperm from the recent mate to reach the sperm-storage organs and displace any resident sperm from the previous mate(s) [17,37,40–42]. The timing of mating plug ejection is thus among the best known mechanisms of postcopulatory sexual selection in *D. melanogaster* and an intrinsic source of sexual conflict between a female and each of her mates over paternity [1,8]. Recent investigations using isogenic lines of *D. melanogaster* have demonstrated significant additive genetic variation for plug ejection time [41,42] and significant female × male genotypic and phenotypic interactions on plug ejection time [42].

To test the prediction that the FRT contributes to molecular female × male interactions underlying the formation of the mating plug of *D. melanogaster* and the timing of its ejection, we used a historical *Lozenge* mutant that impacts development of the exocrine glands of the FRT (i.e. the spermathecae and parovaria) [43] and quantitative proteomics to examine integration of secreted FRT glandular proteins into the mating plug. The extensive characterization of male seminal fluid [44] and sperm [45,46] proteomes along with recent investigations of the FRT transcriptome [47] and proteome [48] allowed us to identify female contributions to molecular female × male interactions underlying a known target of intense sexual selection and a source of sexual conflict.

## 2. Methods

### (a) Fly strains and maintenance

**Figure 1.**
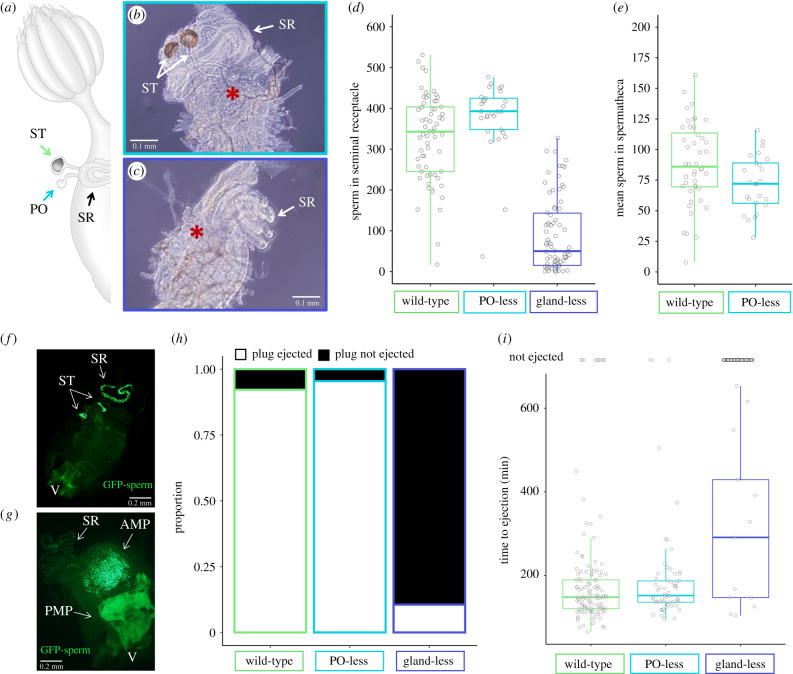
Mutant *Lozenge* alleles result in abnormal FRT gland development, reduced sperm storage and retention of the mating plug. (*a*) Schematic of the wild-type female FRT. (*b*) Parovaria-less females had spermathecae and seminal receptacle that did not appear to differ from wild-type. Star indicates the base of the spermathecae ducts where there is no evidence of the corresponding parovaria ducts. (*c*) Gland-less females had a seminal receptacle that appeared like wild-type but there was no evidence of spermathecae or parovaria ducts on the ventral side (marked with a star). (*d*) Number of sperm stored in the seminal receptacle was significantly reduced in gland-less females but not in females without parovaria. (*e*) There was no significant difference in the number of sperm stored in the spermathecae of FRTs without parovaria. (*f*) Representative wild-type female approximately 24 h after mating. (*g*) Representative gland-less female approximately 24 h after mating. The mating plug can be seen in the FRT, the anterior portion (AMP) is predominantly sperm and the ejaculate protein dense posterior portion (PMP) was used in the proteomic analysis. (*h*) Gland-less females had a significantly higher proportion of females that did not eject the plug within 24 h postmating (*i*) Wild-type and parovaira-less females ejected the mating plug within 3 h. The few gland-less females which did eject the plug took on average twice as long, although the time was highly variable. Abbreviations: anterior mating plug (AMP), parovaria (PO), posterior mating plug (PMP), seminal receptacle (SR), spermathecae (ST) and vagina (V). (Online version in colour.)

Three lines of *D. melanogaster* with different *Lozenge* mutations were used (provided by the Bloomington Drosophila Stock Center; BDSC): lz^1^ (lz^1^/FM3; BDSC #63), lz^3^ (lz^3^/C^1^DX,y^1^f^1^; BDSC #64) and lz^s^ (C^1^DX,y^1^f^1^/ln(1)dl-49,w^1^lz^s^; BDSC #4040). With these strains, we generated flies with three different FRT phenotypes: (i) ‘wild-type' sibling control females (possess both glands, parovaria and spermathecae; lz^1^/+ and lz^s^/+), (ii) ‘parovaria-less' females (lack only parovaria; lz^3^/+), and (iii) ‘gland-less' females (lack both spermathecae and parovaria; lz^1^/ lz^1^, lz^1^/ lz^3^ and lz^1^/ lz^s^) (figure 1*a*-*c*). *Lozenge* expression has previously been shown to be restricted, in the genital disc and FRT, to the glandular cells and their precursors [49]; however, we cannot eliminate the possibility that these mutations influence other FRT tissues indirectly. We additionally used standard wild-type strain (LH_M_) females as a second control and LH_M_ males for approximately half of the matings. The other half of matings were with males that had green fluorescent protein (GFP) tagged protamine B making individual sperm heads distinguishable and readily quantifiable [37]. All flies were maintained on a standard cornmeal-molasses media supplemented with live yeast at approximately 22°C and 12 L : 12 D cycle). Experimental females were collected within 12–14 h of eclosion and housed in single-sex vials of approximately 10 flies with media and live yeast for 3–5 days prior to experiments. Vials were checked for larvae to ensure that only unmated females were used for experiments.

### (b) Ejection timing and sperm counts

Females of all genotypes were mated individually in vials with media to either standard LH_M_ or GFP-sperm males over four experimental trials and the duration of copulation was recorded. Within 1 h postmating, each female was transferred to a respective 6.5 mm-deep well in a glass plate beneath a glass coverslip. Each well contained a drop of 5% apple juice-agar (with black food colouring), which prevented desiccation of females and enhanced visibility of ejected plugs. Wells were examined for an ejected plug at 15 min intervals for a period of at least 6 h postmating, by which time sperm ejection typically occurs in *D. melanogaster* [37,41,42]*.* For females not observed to have ejected the plug during that time (or if plug identification was ambiguous), wells were further examined at 9, 12 and 24 h postmating. Females were frozen at −80°C shortly after plug ejection, or at 24 h postmating if no ejection was observed.

Each frozen female was thawed and the lower FRT (i.e. intact FRT excluding the lateral oviducts and ovaries) was dissected intact in a drop of phosphate-buffered saline (PBS). Presence/absence of spermathecae and parovaria was recorded as well as whether auto fluorescent mating plug material was visible in the bursa. If the presence of the plug was inconsistent with ejection observations, the observation of the plug superseded the record of observed ejection time, which was less precise (59 discrepancies/393 samples across phenotypes). Inconsistent data were excluded from analyses of ejection timing but included in analysis of whether the plug was ejected by 24 h. For females mated to GFP-sperm males, the number of sperm stored in the seminal receptacle and spermathecae, if present, were quantified at 400× on an Olympus BX60 microscope with a GFP filter (Semrock, Rochester NY). However, sperm was not sufficiently visible to count in the spermathecae for all samples, thereby reducing sample sizes for those analyses. Phenotypic data can be found in the electronic supplementary material, table S1.

### (c) Mating plug sample preparation and mass spectrometry

Vials of 10–15 wild-type or gland-less females were combined with an excess of LH_M_ males. Approximately 1 h after the majority of females were observed in copula, females were removed and stored at −80°C. The lower FRT of females was then dissected and the mating plug isolated as previously described [38]. The anterior region of the plug, which primarily contained sperm, was separated and discarded, and the posterior two-thirds of each plug were rinsed and transferred to a 1.5 ml Eppendorf tube with PBS. Approximately 50 plugs were collected for each sample, which were then washed three times with PBS, combined with 50 µl detergent (1 M HEPES with 2% SDS and 5% β-mercaptoethanol), and solubilized through alternate rounds of heating at 95°C and mechanical disruption with a pellet pestle homogenizer.

Liquid chromatography tandem mass spectrometry (LC-MS/MS) was conducted by Cambridge Proteomics following standard protocols (see [50]). In brief, approximately 30 µg of each sample was reduced, alkylated, trypsin digested and labelled with 6-plex tandem mass tags (TMT, Thermo Scientific). Plugs from wild-type females were labelled with 126, 127, 128 tags and plugs from gland-less females were labelled with 129, 130, 131 tags. Labelled peptides were combined, cleaned and separated for 60 min by high pH reverse-phase chromatography on a C18 column (Acquity UPLC) and combined into 15 fractions. Peptides within fractions were separated by liquid chromatography on a Dionex Ultimate 3000 rapid separation nanoUPLC system (Thermo Scientific) with an Acclaim 100 C18 pre-column (PepMap) and reverse-phase nano EASY-spray column (PepMap) and then sprayed into the Lumos Orbitrap mass spectrometer. Raw spectral files are available from the PRIDE ProteomeXchange Consortium [51] with the identifier PXD028524.

### (d) Protein identification and quantification

Raw spectral data was analysed with Proteome Discoverer v.2.3 (Thermo Fisher Scientific) and Mascot v.2.6 with reference to a database of the longest isoform of the *D. melanogaster* genome (r6.21) [52], excluding common contaminant proteins (cRAP v 1.0; thegpm.org), and allowing for a MS tolerance of ±10 ppm, MS/MS tolerance of ±0.8 Da, and up to two missed tryptic cleavages. Search parameters accounted for fixed protein modification of carbamidomethylation (cysteine) and allowed for variable modifications of oxidation (methionine) and deamidation (glutamine and arginine). Proteins were quantified for each sample as the sum of the centroid TMT tag receptor ions (± 2 millimass unit window) correcting for isotopic label purity.

The proteome had a total of 657 832 MS/MS spectra which corresponded to 20 962 peptide spectral matches (PSMs) and the identification of 1508 high-confidence proteins (false discovery rate ≤ 0.01, two unique peptides, and present in every sample). Protein intensities for each sample were log transformed and median normalized in MSnbase [53] and significant differential abundance was calculated with empirical Bayes moderated *t*-tests (Benjamini-Hochburg correction for multiple comparisons) using LIMMA [54]. Normalized proteomic data can be found in the electronic supplementary material, table S2. Departures from parity in the number of differentially abundant proteins between mating plugs from FRTs with and without glands were calculated with a weighted binomial test. A non-parametric Kruskal-Wallis test was used to compare differences in mean protein abundance or changes in protein abundance between categories of proteins. Gene ontology (GO) enrichments of differentially abundant proteins were analysed with DAVID using the entire plug proteome as a background [55]. GOs were considered significantly enriched if the Bonferonni adjusted *p-*value was less than 0.05. All significant GO categories from analyses can be found in the electronic supplementary material, table S3.

Proteins were categorized as seminal fluid proteins (SFPs) if they were present in the recent catalogue of curated high confidence or candidate SFPs [44] or if they had both significantly male-biased reproductive tract expression (adjusted *p* < 0.05 and log2 fold change (FC) > 2) relative to the FRT and significantly male reproductive tract biased expression (adjusted *p* < 0.05 and log2 FC > 2) relative to whole male body (read counts from [56] for w118 and Oregon-R strains analysed with DEseq2 [57]; electronic supplementary material, tables S2 and S4). Identification of sperm proteins was based on presence in the sperm proteome [45,46]. Proteins curated as both SFPs and sperm proteins were categorized only as SFPs. Proteins were considered female-derived if they were identified in the FRT transcriptome or proteome characterized in LH_M_ females [47,48]. Patterns of gene expression in FRT tissues were visualized with a heatmap using log2 normalized counts per million (CPM) and tissue-specificity was determined as described in [47]. All external data sources used in these analyses are listed in the electronic supplementary material, table S5. We note that gene expression may vary across strains used in other studies.

### (e) Statistical analysis and data availability

We analysed the influence of FRT phenotypes on postmating traits using mixed effects generalized linear models with FRT phenotype as a fixed effect and random effects of female genotype nested in female phenotype, male genotype (excluding analyses of sperm number) and trial [58]. Inclusion of random effects was determined by stepwise comparison of models with single term deletions that had the lowest variance with a Pearson *χ*^2^ test. Final models for analyses were (i) copulation duration: copulation time min ∼phenotype + (1|trial), Gaussian family (ii) number of sperm stored in the seminal receptacle: sperm in seminal receptacle ∼ phenotype + (1|phenotype/female genotype) + (1|trial), Gaussian family, (iii) number of sperm stored in spermathecae: sperm in spermathecae ∼ phenotype, Gaussian family, and (iv) mating plug ejection: plug ejected ∼ phenotype + (1 |trial), binomial model. Comparisons between parovaria-less or gland-less females to sibling controls were corrected for multiple comparisons with the Dunn-Bonferroni correction. For all analyses, the wild-type sibling and LH_M_ females were found to be statistically identical for all variables (*p* > 0.05, data not shown), supporting that the *Lozenge* sibling controls have functionally normal parovaria and spermathecae. Data were analysed with R, version 6.3 [59] and visualized with ggplot2 [60]. Data summaries are presented as mean ± 1 s.e.

## 3. Results

### (a) Female reproductive tract glands are necessary for sperm storage

We generated allelic combinations of *Lozenge* mutants which, consistent with their initial description [43], resulted in the absence of either the parovaria or both FRT glands (i.e. the parovaria and spermathecae). No *Lozenge* mutants affected development of only the spermathecae. In both the gland-less and parovaria-less phenotypes all the glandular tissue, including the ducts, were consistently absent (figure 1*a–c*). Using these phenotypes, we investigated the effect of FRT gland loss on copulation duration, sperm storage, and mating plug ejection. In contrast to the original characterization of these mutants [43], FRT phenotype was not significantly correlated with variation in copulation duration (*p* = 0.34; electronic supplementary material, figure S1). We did, however, confirm the ascribed role of FRT glands in sperm storage [43], with significant effect of FRT phenotype on number of sperm in the seminal receptacle (*p* = 0.015) but not the spermatheca (*p* = 0.08). In particular, significantly fewer sperm were stored in the seminal receptacle of gland-less females (88.6 ± 10.3 sperm, *n* = 78; *p* = 0.04) compared to wild-type females (327.6 ± 13.3 sperm, *n* = 63; figure 1*d*). Parovaria-less females did not differ significantly from the wild-type with respect to sperm storage in the seminal receptacle (377.1 ± 16.8 sperm, *n* = 29; *p* = 0.7) or spermatheca (72.2 ± 4.6 sperm, *n* = 25 parovaria-less females and 88.2 ± 5.1 sperm, *n* = 44 wild-type females; *p* = 0.54; figure 1*e*). These results indicate that FRT glands, but not the parovaria alone, influence the number of sperm stored in the seminal receptacle. This is consistent with previous studies which demonstrate that FRT glands are necessary for proper sperm storage maintenance and female fertility [43,61–63].

### (b) Female reproductive tract glands influence mating plug ejection

While characterizing mutant phenotypes, we observed that sperm storage differences were associated with retention of the mating plug and sperm presence in the bursa (figure 1*f,g*). To quantify the effect of FRT glands on mating plug ejection, we tracked the occurrence of mating plug ejection within a 24 h period and observed a significant effect of FRT phenotype on plug ejection (*p* < 0.001). Whereas the majority of wild-type females (92.2%, *n* = 128) and parovaria-less females (95.5%, *n* = 66) ejected the mating plug within 24 h, most gland-less females retained the plug for 24 h (10.6% eject, *n* = 180; *p* < 0.001; figure 1*h*). Moreover, for those gland-less females that did eject the plug, the mean ejection time (311.9 ± 55.5 min, *n* = 13) was highly variable and, on average, approximately twice that observed for wild-type females (163.1 ± 6.3 min, *n* = 107) or parovaria-less females (172.0 ± 9.3 min, *n* = 55; figure 1*i*). We therefore conclude that lack of FRT glands, and most likely the spermathecae specifically, plays a functional role in mating plug ejection and sperm storage. However, the parovaria and spermatheca may have redundant functionality and we therefore cannot formally rule out parovaria contributions given the mutant phenotypes available for analysis. We also cannot eliminate the possibility that *Lozenge* mutations (or the absence of glands throughout development) have an indirect effect on other FRT tissues which contributes to the observed phenotypes.

### (c) Mating plug proteome composition

To evaluate whether the absence of glandular tissues influenced mating plug composition, we conducted high throughput mass spectrometry proteomic analyses on mating plugs from wild-type and gland-less females. We robustly identified 1508 proteins (electronic supplementary material, table S2) with high levels of reproducibility between biological replicates (*r* > 0.95; electronic supplementary material, figure S2). Wild-type mating plug protein identification was consistent with a previous proteomic characterization, including 78.5% of the 65 mating plug proteins previously identified [38]. We also identified 172 of the 292 (58.9%) curated SFPs and 108 of 309 (35.0%) candidate SFPs [44] (electronic supplementary material, figure S3A). In addition, we identified 37 putative novel SFPs (see Methods for identification criteria; electronic supplementary material, table S4). These 317 SFPs were significantly higher in abundance than the remainder of plug proteins in both the wild-type and gland-less female (wild-type: Kruskal–Wallis *χ*^2^ = 196.2, *p* < 0.001; gland-less: Kruskal–Wallis *χ*^2^ = 101.1, *p* < 0.001; electronic supplementary material, figure S3B) and comprised 64.6% of the total protein in the wild-type mating plug proteome (electronic supplementary material, figure S3C). Among the most abundant SFPs were *Acp36DE, Ebp, EbpII, Acp53C14a* and *Acp53c14b*, all of which have been identified as predominant plug components that influence sperm storage and/or female re-mating [38,64–68].

Although SFPs constituted the majority of the mating plug contents, they represented only 21% of the identified proteins (electronic supplementary material, figure S3D). We also identified 582 non-SFP sperm proteins [45,46]. Unexpectedly, the majority of mating plug proteins (1120 proteins, 74.3% of total identified proteins) were potentially female-derived, based on identification in recent analyses of the FRT transcriptome [47] and proteome [48]. These female-derived proteins tended to be lower in abundance in the mating plug than ejaculate proteins (electronic supplementary material, figure S3B). Notably, these categories are not mutually exclusive and sex-of-origin cannot be definitively ascribed in many cases as putative SFPs or sperm proteins also exhibit evidence of expression in the FRT (electronic supplementary material, figure S3C,D). Thus, the composition of the mating plug indicates that it is a complex combination of male- and female-derived proteins.

### (d) Female reproductive tract glands contribute proteins to the mating plug

**Figure 2.**
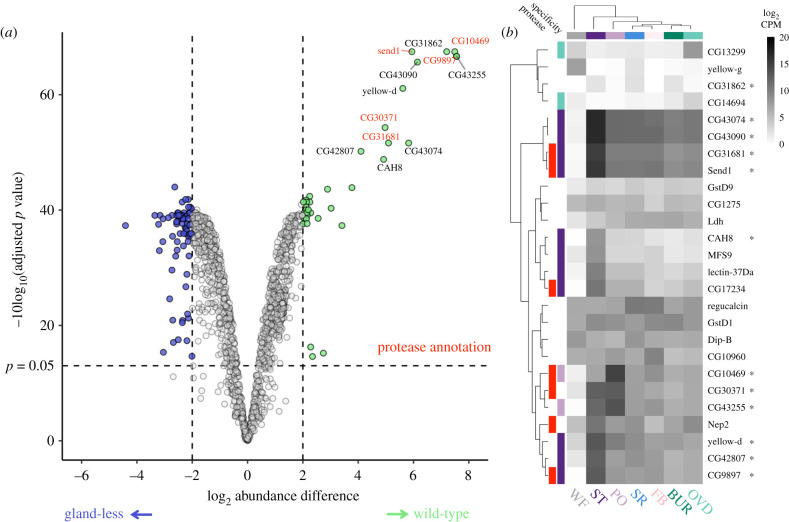
Significant differences in mating plug proteome from gland-less females. (*a*) Volcano plot of abundance differences between mating plugs from wild-type females and gland-less females. Proteins significantly more abundant (adj. *p* < 0.05 and log2FC > 2) from the gland-less females are in blue (left) whereas those more abundant from the wild-type females are in green (right). The 12 highly differentially abundant proteins with log2FC > 4 in the wild-type female plug were labelled by name, with endopeptidases in red. (*b*) Heatmap of FRT tissue gene expression for proteins that had significantly greater abundance in the wild-type female plug. Log2 counts per million (CPM) expression from McDonough-Goldstein *et al*. [47] is shown for each FRT tissue with complete Euclidian clustering for both columns and rows. Annotation columns show whether a gene had an endopeptidase annotation and/or tissue-specific expression. Half of proteins had either spermathecae- or parovaria-specific expression. Stars indicate the 12 proteins with a log2FC > 4 in wild-type female mating plugs. Abbreviations: bursa (BUR), FRT-associated fat body (FB), oviduct (OVD), parovaria (PO), seminal receptacle (SR), spermatheca (ST), whole female without FRT (WF). (Online version in colour.)

We next analysed mating plug proteomic differences between wild-type and gland-less females and identified 111 differentially abundant proteins (7.3% of the plug proteome; adj. *p* < 0.05 and log2FC > 2; figure 2*a*). There were significantly fewer proteins with greater abundance in the wild-type female plug (33.4%, 36/111 differentially abundant proteins; cumulative binomial *p* < 0.001). However, these 36 proteins exhibited a substantially greater magnitude of change, with a log2FC greater than 4 in one third (12 out of 36 proteins) of proteins (compared to 1 out of 75 with a log2FC > 4 in the gland-less female mating plug; cumulative binomial *p* < 0.001). Proteins with greater abundance in the wild-type female plug were enriched for having a secretion signal (UP Keyword Signal *p* = 0.0004) and serine-type endopeptidase activity (GO: 0004252, *p =* 0.01; electronic supplementary material, table S3). Among these proteins were the serine proteases *send1* and *CG9897,* which have been shown to influence female remating rate [69]. We examined the FRT gene expression profiles of these proteins to evaluate if they are representative of glandular secretions and found that 72.2% (26 out of 36 proteins), including all 12 of the proteins with log2FC > 4, were expressed in FRT tissues [47]. Moreover, 50% (13 out of 26 genes with greater abundance in the wild-type female mating plug) had tissue-specific expression in either the spermathecae or parovaria, consistent with expected expression profile of glandular secretions (figure 2*b*). Three of these proteins, *CG43090, CG43074, CG42807,* appear to be exclusively female-derived and were probably previously mischaracterized as SFPs [38,44,47]. Thus, FRT glands appear to contribute proteins to the mating plug, including numerous proteolytic enzymes, and this may be mechanistically related to proper plug ejection.

By contrast, proteins exhibiting greater abundance in the mating plug from the gland-less female exhibited more modest levels of change. The majority of these proteins were identified as female-derived (70 out of 75 proteins with greater abundance in the gland-less female mating plug). However, only 18.5% (12 out of 75 proteins) of these proteins had FRT tissue-specific gene expression profiles, including six with bursa-specific expression and four seminal receptacle-specific expression. The lack of tissue-specific expression suggests that secretions from other FRT tissues have a greater relative representation in the plug composition in the absence of glandular proteins. Proteins more abundant in the gland-less female mating plug were enriched for membrane components (GO:0016021, *p* = 0.004) and constituents of the cuticle (GO:0042302, *p* = 0.04; electronic supplementary material, table S3), which is consistent with holocrine or apocrine secretion of intracellular and structural proteins into the FRT lumen [48]. However, we cannot distinguish whether these proteins are the result of non-specific associations of the mating plug with FRT luminal fluid or whether they may represent female responses, perhaps immunological, to prolonged presence of the mating plug.

### (e) Female reproductive tract glandular tissues influence male-derived mating plug composition

**Figure 3.**
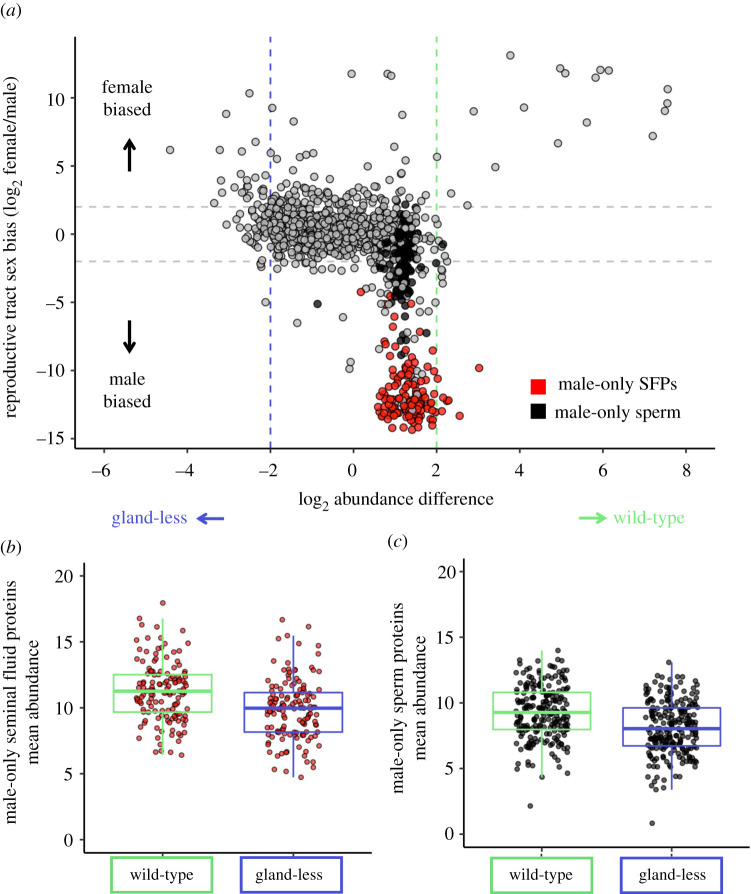
Sex-biased expression of mating plug proteins. (*a*) Sex-biased gene expression (female/male reproductive tract; log2FC > 2) from Yang *et al*. [56] indicates that male-biased proteins tended to have greater abundance in the wild-type female mating plug. The majority of male-biased proteins were either SFPs (red) or non-SFP sperm (black). Male-only (*b*) SFPs or (*c*) non-SFP sperm proteins exhibited significantly higher abundance on average in mating plugs from wild-type females. (Online version in colour.)

To further delineate between male and female contributions to the mating plug, we examined abundance differences with respect to sex-biased gene expression in the reproductive tract (figure 3*a*) [56]. We found no significant difference in the abundance of proteins encoded by genes with female-biased expression (mean log2FC = −0.27; Kruskal–Wallis *χ*^2^ = 0.77, *p* = 0.40). However, for proteins with male-biased expression protein (abundance was significantly higher in wild-type female mating plugs (mean log2FC = −1.09; Kruskal–Wallis *χ*^2^ = 27.26, *p* < 0.001). A direct comparison of male-derived (i.e. no FRT tissue expression [47]) SFPs and sperm proteins revealed that these proteins had significantly greater abundance in plugs from wild-type than from gland-less females (SFP: Kruskal–Wallis *χ*^2^ = 22.68, *p* < 0.001; figure 3*b*; sperm: Kruskal–Wallis *χ*^2^ = 30.4, *p* < 0.001; figure 3*c*). The greater abundance of putative ejaculate proteins in a wild-type FRT environment predicted to have higher proteolytic activity that could degrade ejaculate proteins, leads us to hypothesize that glandular tissues, and most likely their secreted protein products, regulate processes governing mating plug formation.

## 4. Discussion

The mating plug is an essential element of many mating systems and has independently arisen in diverse taxa, including nematodes, acathocephalan worms, insects, arachnids, reptiles and mammals [31]. Plug formation and persistence are expected to be targets of intense sexual selection (including sexual conflict) [31]. Indeed, the rate of evolutionary diversification of male genes critical to plug formation has been shown to correspond with variation in the intensity of postcopulatory sexual selection among primate species [70]. Furthermore, the timing of plug ejection by *D. melanogaster* females significantly contributes to variation in competitive fertilization success [37,40–42] and reproductive isolation between sister species [40]. The initial proteomic analysis of the *D. melanogaster* mating plug focused exclusively on male-derived SFPs within the plug [38]. Hence, little is known about the molecular basis for female contributions to the genotypic and phenotypic female × male interactions that influence plug ejection [41,42], although the female neurological system has been implicated in this process [71]. Our examination of the *D. melanogaster* mating plug demonstrates that the absence of FRT glands is associated with changes in the composition of the mating plug and the timing of ejection. We thus hypothesize that FRT glandular secretions contribute to processes responsible for mating plug dynamics.

In comparing the mating plug between wild-type and gland-less females, we identified candidate FRT glandular secretions that may influence plug function. FRT secretions have previously been hypothesized to contribute to the digestion and/or ejection of the mating plug and other ejaculate structures. Specifically, the expression of female-derived proteolytic enzymes and inhibitors in the mating plug have been hypothesized to represent a molecular mechanism of sexual conflict over the timing of plug degradation [31]. Proteolytic activity in the FRT has been enzymatically demonstrated in the cabbage white butterfly, *P. rapae* [72], and in two *Drosophila* species (*Drosophila arizonae* and *Drosophila mojavensis*) [73], where it has been associated with breakdown of the spermatophore and the ejaculate-induced ‘insemination reaction', respectively. In addition, glandular secretion of peptidases in the mouse are involved in the digestion of ejaculate proteins as part of the process of semen liquefaction, an event critical for enabling sperm to move through the FRT [74]. Here, we present molecular data that expands the scope of female involvement in interactions that influence mating plug dynamics. We demonstrate that FRT glands have a widespread impact on the proteomic composition of the mating plug as a potential mechanism through which the female may influence the processes responsible for formation and ejection of the plug. Future experiments to confirm the role of female glandular secretions in mating plug dynamics, and the specific contributions of the spermatheca or parovaria, could include rescue experiments through the induction of specific glandular protein expression, tissue-specific knockdowns of candidate female mating plug proteins, and visualization of tagged female proteins to assess spatio-temporal mating plug associations.

The influence of FRT glandular proteins on plug formation and ejection may occur through three non-mutually exclusive mechanisms. First, female secretions may be present during plug formation and become integral plug components, as has been shown to occur with the spermatophore of the butterfly, *P. rapae* [32]. Second, female secretions may interact with seminal fluid constituents and regulate the formation and composition of male-derived proteins in the plug. Third, female secretions may interact with the fully-formed plug and contribute to its processing or degradation. Our proteomic analyses of the plug, supported by our recent transcriptomic [47] and proteomic [48] investigations of the *D. melanogaster* FRT, provide empirical support for the first hypothesis. Specifically, we found numerous abundant plug components with expression profiles in a wild-type strain indicative of female glandular secretions, suggesting that female proteins are incorporated into the plug during formation. With respect to the greater abundance of putatively male-derived plug components in wild-type females, we cannot formally distinguish between the second and third mechanisms of formation and degradation based on our analysis of a single timepoint. However, the observed pattern is not consistent with a simple model of FRT secretions contributing to plug degradation at one-hour postmating because the mating plug in a wild-type female has a greater abundance of female-derived proteases. As such, the second mechanism, in which the absence of female-derived proteins in the gland-less female results in the dysregulation of plug formation and a widespread, significant reduction of male-derived SFPs and sperm incorporated into the mating plug, is more parsimonious. Proteomic analyses of the mating plug across multiple timepoints would be required to characterize the processes of plug formation and degradation to distinguish between these alternatives. We also note, that we cannot rule out the possibility that the absence of female glandular tissues somehow influences male ejaculate tailoring [75–77]. Finally, as we demonstrate that FRT glands are necessary for sperm ejection, we predict that the plug composition changes in the gland-less female, are ultimately associated with a disruption in the processes of plug degradation that result in ejection.

In conclusion, we demonstrate a novel function of the spermathecae and parovaria in the timing of sperm ejection and characterize an important molecular mechanism through which females may influence mating plug composition and the timing of sperm ejection. The influence of female glandular tissues not only on putative female secretions but also ejaculate components of the plug is indicative of how sex-specific changes can influence male × female interactions to impact female fitness and differential male reproductive success [37,41,42]. We hypothesize that regulation of the extracellular FRT environment by glandular tissues represents an important and widespread molecular mechanism of postcopulatory sexual selection in diverse animal taxa. This hypothesis is supported by recent studies demonstrating variation in *Drosophila* FRT gene or protein expression between species or in response to the intensity of sexual selection [50,78–80]. However, characterization and functional analyses of female glandular secretions have not been widely pursued (but see in insects [81–83]). Investigations of FRT secretions, coupled with studies that characterize intraspecific variation in FRT and the interactive consequences for male and female fitness, will greatly advance our understanding of sexual selection, including sexual conflict.

## Data accessibility

Phenotypic data are available in the electronic supplementary material, table S1 [84]. Proteomic data are available as raw spectral reads on the ProteomeXchange (PXD028524) and protein abundance and quantitation in the electronic supplementary material, table S2. Code used to analyse data can be accessed at https://github.com/CEMcDonoughGoldstein/FRTglandMatingPlug.
